# Ultra-rapid prolactin normalization with cabergoline in a premenopausal woman with epilepsy and giant prolactinoma

**DOI:** 10.1210/jcemcr/luag105

**Published:** 2026-04-21

**Authors:** Perrin Ngougni Pokem, Stefan Matei Constantinescu, Hadrien Cools, Dominique Maiter, Damien Gruson

**Affiliations:** Department of Laboratory Medicine, Cliniques Universitaires Saint-Luc, UCLouvain, Brussels B-1200, Belgium; Department of Endocrinology and Nutrition, Cliniques Universitaires Saint-Luc, Brussels B-1200, Belgium; Department of Laboratory Medicine, Cliniques Universitaires Saint-Luc, UCLouvain, Brussels B-1200, Belgium; Department of Endocrinology and Nutrition, Cliniques Universitaires Saint-Luc, Brussels B-1200, Belgium; Department of Laboratory Medicine, Cliniques Universitaires Saint-Luc, UCLouvain, Brussels B-1200, Belgium; Endocrinology, Diabetes and Nutrition Research Centre, Institute for Experimental and Clinical Research, Cliniques Universitaires Saint-Luc and UCLouvain, Brussels B-1200, Belgium

**Keywords:** giant prolactinoma, hyperprolactinemia, cabergoline, epilepsy, biochemical–radiological dissociation

## Abstract

Giant prolactinomas are rare pituitary tumors characterized by extremely high prolactin levels and extensive extrasellar invasion. While dopamine agonists are usually effective, they may require months to normalize prolactin. We report a 47-year-old premenopausal woman presenting with new-onset temporal epilepsy and secondary amenorrhea. Assessments revealed marked hyperprolactinemia (mean serum prolactin concentration, 3902 ng/mL [SI: 169.8 nmol/L]) (reference range, 5.0-30.0 ng/mL [SI: 0.22-1.30 nmol/L]), and an invasive 47 × 43 × 50 mm adenoma extending into the right temporal lobe. After initiating cabergoline (0.25 mg/day), serum prolactin dropped to 802 ng/mL (SI: 34.9 nmol/L) within 18 hours and normalized by day 4 (12.7 ng/mL [SI: 0.55 nmol/L]). Polyethylene glycol recovery excluded macroprolactinemia. Despite ultra-rapid biochemical remission, magnetic resonance imaging at 3 and 6 months revealed no tumor shrinkage despite increasing cabergoline to a maximal tolerated dose of 0.75 mg/week. The patient remained seizure free and resumed normal menses. This case illustrates a striking dissociation between hormonal sensitivity and a lack of radiological regression.

## Introduction

Giant prolactinomas are rare tumors characterized by a maximal diameter above 40 mm, extremely elevated serum prolactin levels (typically above 1000 ng/mL [SI: 43.5 nmol/L], and sometimes reaching >10 000 ng/mL [SI: >435 nmol/L]), male predominance, and extensive extrasellar involvement [1, 2]. Symptoms arise both from hyperprolactinemia and the subsequent inhibition of the gonadotrope axis and from the compression of surrounding structures by the tumor. In addition, giant prolactinomas may initially present with unusual manifestations, such as temporal epilepsy, hydrocephalus, or spontaneous cerebrospinal fluid (CSF) leak [1-3].

Dopamine agonists (DAs) are the primary treatment for prolactinoma, independent of their size, with cabergoline being particularly effective in normalizing prolactin levels and reducing tumor size [4, 5]. Although giant prolactinoma may be more resistant to medical therapy than typical micro- and macroprolactinomas, serum prolactin levels have been reported to normalize in 50-60% of patients. Tumor size decreases significantly in about 70-80% of patients within several months after initiating DA therapy [1-5].

We report the unusual case of a premenopausal woman presenting with temporal epilepsy triggered by a giant prolactinoma, in whom low-dose cabergoline administration induced an ultra-rapid normalization of prolactin levels within 4 days but, in contrast, no tumor size reduction after 6 months of excellent hormonal control.

## Case presentation

**Figure 1:**
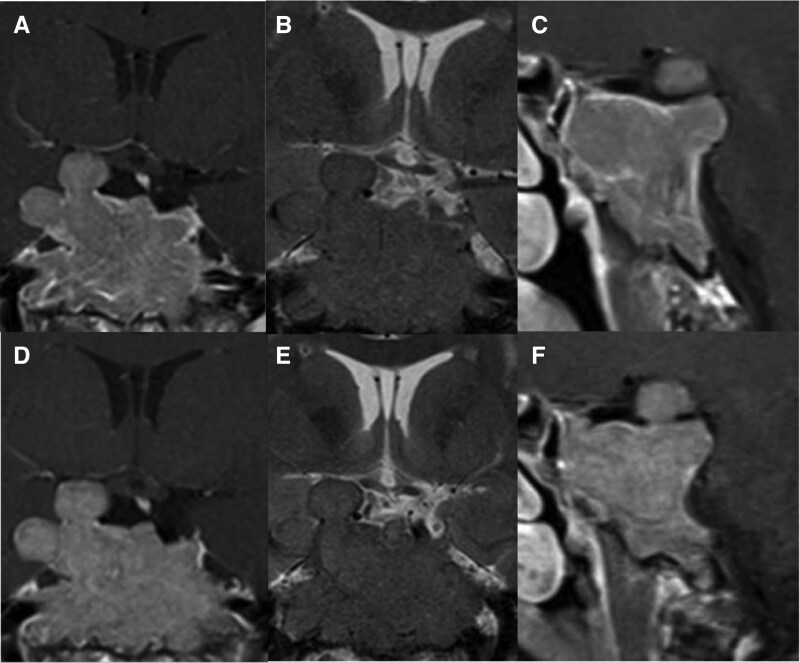
Pituitary MRI at baseline and during follow-up under cabergoline therapy. (A–C) Post-gadolinium T1 coronal (A) and sagittal (C) sections, and coronal T2 (B) sections of the pituitary MRI at diagnosis, showing a giant invasive sellar and parasellar mass with clival, sphenoidal and right temporal extension, compressing the pituitary gland. (D–F) Corresponding sections after 6 months of cabergoline treatment, demonstrating stable tumor size without significant shrinkage despite complete biochemical remission of hyperprolactinemia. This illustrates the dissociation between rapid hormonal normalization and delayed or absent radiological response in giant invasive prolactinomas.

A 47-year-old woman with a history of distal spinal muscular atrophy presented to the emergency department for *de novo* seizures with features suggestive of temporal epilepsy (episodes of blank stare and loss of consciousness with tongue biting, head deviation, and automatisms but without urinary loss or convulsions). Electroencephalogram showed a moderately disturbed pattern due to its lack of structure, with no abnormal epileptiform features. Magnetic resonance imaging (MRI) of the brain was performed to rule out structural abnormalities in the context of *de novo* seizures and revealed a 47 × 43 × 50 mm lobulated mass centered on the sella turcica and the clivus, extending into the nasopharynx, both cavernous sinuses and the right temporal lobe, with a deviation of the pituitary stalk to the left (Fig. 1 A-C). The history revealed secondary amenorrhea since the last 3 years. However, the patient did not report any headache, breast tension, or galactorrhea.

## Diagnostic assessment

Endocrinological assessment showed marked hyperprolactinemia (serum prolactin concentration, 3472 ng/mL [SI: 151.0 nmol/L] (reference range, 5-30 ng/mL [SI: 0.22-1.31 nmol/L]), which was subsequently confirmed by two repeat measurements within 24 hours (4823 ng/mL [SI: 209.8 nmol/L] and 3411 ng/mL [SI: 148.4 nmol/L]; mean of the 3 values, 3902 ng/mL [SI: 169.8 nmol/L]). This was associated with hypogonadotropic hypogonadism: luteinizing hormone (LH) 0.8 U/L (reference range, 1.0-95.0 U/L); Follicle-stimulating hormone (FSH) 8.1 U/L (reference range, 1.7-21.5 U/L); estradiol 6 pg/mL (SI: 22 pmol/L) (reference range, 12-233 pg/mL [SI: 44-855 pmol/L]), and mild hyponatremia (serum sodium concentration, 131 mmol/L (reference range, 136-145 mEq/L), raising suspicion of additional pituitary deficits. However, morning concentrations of cortisol, adrenocorticotropic hormone (ACTH), thyroid-stimulating hormone (TSH), free thyroxine (T4) and free triiodothyronine (T3), as well as insulin-like growth factor-1 (IGF-1) were normal (Table 1).

**Table 1:** Hormonal values measured at diagnosis and after 3 and 6 months of cabergoline treatment

Parameter	At diagnosis	After 3 months	After 6 months	Reference range
TSH	1.87 mUI/L	1.70 mUI/L	1.05 mUI/L	0.27-4.20 mUI/L
Free T3	2.60 pg/mL (4.0 pmol/L)	2.93 pg/mL (4.5 pmol/L)	2.54 pg/mL (3.9 pmol/L)	2.02-4.43 pg/mL (3.1-6.8 pmol/L)
Free T4	1.25 ng/dL (16.1 pmol/L)	1.04 ng/dL (13.4 pmol/L)	1.41 ng/dL (18.1 pmol/L)	0.93-1.71 ng/dL (12.0-22.0 pmol/L)
IGF-1	106.9 ng/mL (14.0 nmol/L)	—	125.3 ng/mL (16.4 nmol/L)	102.0-267.0 ng/mL (13.3-34.9 nmol/L)
LH	0.7 UI/L	—	14.2 UI/L	1-95 UI/L *(premenopause)*
FSH	8.1 UI/L	—	25.0 UI/L	1.7-21.5 UI/L *(premenopause)*
Prolactin	4823 ng/mL (209.7 nmol/L)	1.4 ng/mL (0.061 nmol/L)	0.4 ng/mL (0.017 nmol/L)	5.0-30.0 ng/mL (0.22-1.30 nmol/L)
Estradiol	6 pg/mL (22 pmol/L)	—	22 pg/mL (81 pmol/L)	12-233 pg/mL (44-855 pmol/L)
Cortisol	18.3 µg/dL (505 nmol/L)	11.0 µg/dL (303 nmol/L)	4.1 µg/dL (113 nmol/L)	4.7-18.1 µg/dL (130-500 nmol/L)
ACTH	16.6 pg/mL (3.7 pmol/L)	—	6.1 pg/mL (1.3 pmol/L)	5-50 pg/mL (1.1-11.0 pmol/L)

Values expressed in conventional units followed by Système International (SI) units in parentheses.

Abbreviations: ACTH, adrenocorticotropic hormone; FSH, follicle-stimulating hormone; IGF-I, insulin-like growth factor I; LH, luteinizing hormone; T3, triiodothyronine; T4, thyroxine; TSH, thyroid-stimulating hormone.

Baseline ophthalmological examination showed normal visual fields and normal optic coherence tomography (OCT).

Although the diagnosis of giant prolactinoma was indubitable, we also excluded the additional presence of macroprolactin. All serum samples underwent precipitation with polyethylene glycol (PEG), following the classical protocol [6]. Serum total and unbound prolactin concentration were measured using automated electrochemiluminescence immunoassay (ECLIA, Roche Diagnostics, Elecsys II prolactin, module 602, USA, Cat# 03203093 190, RRID:AB_2883976).

## Treatment

The patient was admitted in the endocrinology unit for treatment initiation and close surveillance. Levetiracetam was introduced at a dose of 500 mg twice daily for epileptic seizures. Cabergoline was also initiated at a dose of 0.25 mg/day at bedtime for 5 consecutive days, followed by twice-weekly administration thereafter. We performed serial cortisol and ACTH measurements over 24 hours on the day after her admission to exclude partial corticotrope insufficiency. We later also measured prolactin on these samples to better understand the prolactin evolution over time.

## Outcome and follow-up

**Figure 2:**
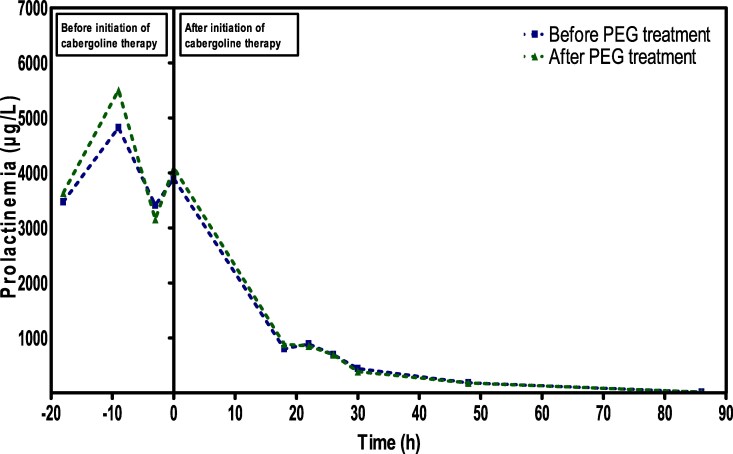
Evolution of serum prolactin concentrations before and after initiation of cabergoline. The figure shows both profiles of total prolactin (green line) and unbound prolactin after polyethylene glycol (PEG) precipitation (blue line). The y-axis corresponds to time 0, marking the initiation of cabergoline therapy. Serum prolactin levels showed a rapid decline, falling from a mean baseline value of 3902 ng/mL (SI: 169.8 nmol/L) to 802 ng/mL (SI: 34.9 nmol/L) within 18 hours, 181 ng/mL (SI: 7.9 nmol/L) at 48 hours, and reaching complete normalization by day 4 (12.7 ng/mL [SI: 0.55 nmol/L]). PEG recovery ranged from 92% to 113%, excluding macroprolactinemia.

As shown in Fig. 2, a dramatic reduction of prolactin (PRL) concentrations was observed within hours after initiation of cabergoline, from a mean pre-treatment value of 3902 ng/mL (SI: 169.8 nmol/L) to 802 ng/mL (SI: 34.9 nmol/L) after 18 hours, confirming the high efficacy of the treatment. At 48 hours, serum prolactin concentration had markedly decreased to 181 ng/mL (SI: 7.9 nmol/L) and was completely normalized by day 4 (12.7 ng/mL [SI: 0.55 nmol/L]). After PEG precipitation, there was no significant difference between total and unbound prolactin, with recoveries ranging from 92% to 113% (Fig. 2).

The episode of hypo-osmolar hyponatremia was corrected within 24 hours of fluid restriction (750 mL/day). The patient was discharged with low doses of cabergoline (0.25 mg twice a week). She was warned of the possibility of tumor apoplexy or CSF leak that could occur under cabergoline treatment, and instructed to present to the emergency department in case of intense headaches, vision disturbances, or rhinorrhea. Regular menses resumed after 3 months of treatment. Serum prolactin concentrations remained fully suppressed at 3.1 ng/mL (SI: 0.13 nmol/L) and 0.4 ng/mL (SI: 0.017 nmol/L) after 3 and 6 months, respectively. However, despite this rapid and excellent hormonal response, MRI follow-up after 3 and 6 months revealed stable tumor dimensions (Fig. 1 D-F), despite up-titration of the cabergoline dose after 3 months up to a maximal tolerated dose of 0.75 mg/week, with no further effect on tumor size. The patient suffered from dizziness on 1 mg/week of cabergoline and could not tolerate more than 0.75 mg/week. Hydrocortisone 10 mg in the morning was initiated at 6 months because of low morning serum cortisol concentration (4.1 µg/dL [SI: 113 nmol/L]) (reference range, 4.7-18.1 µg/dL [SI: 130-500 nmol/L]) and adrenocorticotropic hormone (ACTH) level (6.1 pg/mL [SI: 1.3 pmol/L]) (reference range, 5.0-50.0 pg/mL [SI: 1.1-11.0 pmol/L]) indicating partial ACTH deficiency. Neuro-ophthalmological evaluation at 6 months with visual fields revealed slight hemianopsia on the right eye, but normal OCT, possibly related to the persistent contact of the tumor with the right optic tract.

Debulking surgery was proposed to the patient due to the lack of radiological response and the persistent compression of the temporal lobe and optic nerve. The patient refused surgery because of her stable clinical state on medical treatment and the fear of surgical complications that would further increase her dependency in daily life activities. Indeed, she was already suffering from distal spinal muscular atrophy and partially dependent on her family. Analyses of the aryl hydrocarbon receptor-interacting protein (*AIP*) and multiple endocrine neoplasia type 1 (*MEN1*) genes revealed no pathological variant.

## Discussion

We report the unusual case of a premenopausal woman with temporal epilepsy as the first manifestation leading to the diagnosis of an invasive giant prolactinoma. This tumor showed an exceptionally rapid biochemical remission under very low doses of cabergoline but no radiological shrinkage.

Giant prolactinomas are exceptionally diagnosed in premenopausal women, and this may be explained by several reasons. First, these giant tumors occur much more frequently in men, with a male-to-female ratio of 9:1 [7]. In addition, most female cases are diagnosed at an older age, usually on the basis of tumor pressure symptoms, although endocrine symptoms such as amenorrhea are present but overlooked for a long period of time [7].

Among the various symptoms that can be caused by these tumors, epileptic seizures are a rare presenting feature of prolactinomas. Transient postictal hyperprolactinemia may occur in epilepsy without pituitary disease, but levels usually remain below 100 ng/mL (SI: 4.35 nmol/L) and normalize within hours [8]. In contrast, our patient presented with persisting prolactin concentrations far beyond the range of seizure-related hyperprolactinemia and fully consistent with a giant prolactinoma [1, 2, 5, 9]. Temporal epilepsy is the most frequent type observed in such conditions and is characterized by focal seizures starting with an aura and progressing to periods of impaired awareness. Of note, other common causes of mild-to-moderate hyperprolactinemia (pharmacological agents, systemic disease) were also excluded, and no significant level of macroprolactin was found after PEG precipitation [4, 5, 10].

In large tumors, cabergoline typically leads to a progressive biological response with a median time to prolactin normalization reported at 25 months in a recent series of giant prolactinomas [11]. In this case, serum prolactin dropped very rapidly, normalized within just 4 days and remained completely suppressed on a very low-dose regimen (0.25 mg/day for 5 days, then 0.25 mg twice weekly). Such rapid normalization is exceptional and reflects an exquisite sensitivity of the tumor to cabergoline, at least in terms of prolactin secretion. Starting with a very low dose of DA and gradually up-titrating the dose was consistent with current recommendations of treatment for large invasive tumors, both to improve tolerability and to minimize the risk of apoplexy or CSF leak [4, 12, 13]. Further cabergoline dose escalation might have induced a decrease in tumor diameter but our patient suffered from dizziness and could not tolerate more than 0.75 mg/week of cabergoline.

Another striking feature of this case is the dissociation between the biochemical and radiological responses to DA. Despite rapid and complete normalization of prolactin within days, MRI showed stable tumor size at 6 months despite a slight increase in the cabergoline dose. This phenomenon has been described in series of giant prolactinomas, where a minority of patients achieve endocrine control without significant tumor shrinkage [11, 14]. This dissociation implies that post dopaminergic receptor pathways involved in the anti-proliferative effects are separated from anti-secretory mechanisms [5]. Cabergoline binding to the D2 dopamine receptor decreases prolactin levels by inhibiting the release of prolactin from secretory granules through inactivation of voltage-gated calcium channels and by reducing prolactin gene expression through activation of adenylyl cyclase activity [15]. D2 receptor activation also stimulates the PI3K (phosphatidylinositol 3-kinase) and MAPK (mitogen-activated protein kinase) pathways to inhibit cellular proliferation [15].

The absence of histological characterization and molecular analysis of the tumor clearly represents a limitation of our study. It would have been interesting to further analyze the granulation pattern, proliferation indexes as well as the molecular biology of this unusual tumor, to exclude the rare occurrence of a Pit-1 immature tumor [16]. Pituitary MRI showed the typical features of giant prolactinomas, with a lobulated invasive mass, relatively homogeneous, without major cystic or necrotic components, which was iso-intense both in T1 and T2 sequences, thus without the T2 hypersignal characteristic of chordoma [17]. During follow-up, the patient developed early signs of right-sided hemianopsia, requiring close monitoring. Should visual compromise progress, surgical decompression might be considered, particularly to relieve optic tract compression.

## Learning points

- Giant prolactinomas in premenopausal women are exceptional, and the restoration of regular menstrual cycles after treatment highlights the preserved gonadal axis responsiveness despite the tumor's size and invasiveness.
- Epileptic seizures, particularly of temporal origin, may represent the first manifestation of a giant prolactinoma. This emphasizes the need to distinguish seizure-related transient prolactin elevations from genuine pathological hyperprolactinemia.
- An ultra-rapid biochemical response to cabergoline can occur, leading to normalization of prolactin levels within days. Such a kinetic profile was rarely documented in giant prolactinomas.

## Contributors

All authors made individual contributions to authorship. P.N.P. and S.M.C. were involved in the diagnosis and management of the patient. P.N.P., S.M.C., H.C., D.M., and D.G. analyzed the data and interpreted the results. D.G. and D.M. supervised the work. P.N.P wrote the draft of the manuscript. All authors reviewed and approved the final draft.

## Data Availability

Original data generated and analyzed during this study are included in this published article
